# Process for the Detection of Arsenic

**Published:** 1845-05

**Authors:** H. Letherby

**Affiliations:** Lecturer of Chemistry at the Medical School of the London Hospital


*Process for the Detection of Arsenic.* By H. Letherby, M. D., Lecturer of Chemistry at the Medical School of the London Hospital.—Of the many processes which have from time to time been suggested, and more or less lauded, too, for the detection of arsenic in cases of poisoning, I am confident that but few of them have been put to the test of experiment with organic mixtures, by way of determining their applicability and delicacy, and the manipulator who is accustomed to seek for arsenic, often in very small quantity, in the contents of the stomach, or in the tissues of poisoned animals, has learned to look with distrust upon every new suggestion, relying more upon his own tact with regard to some particular process to which he is accustomed, than caring to re-educate himself by adopting any new one. I put forward, however, the following observations, by reason of some little experience in this department of chemistry, and I am convinced, to speak in a general way, that the apparatus, described in The Lancet of February 1st, and which was proposed by Berzelius, for the detection of arsenic in cases of poisoning, is not at all applicable, and more particularly in the hands of a novice; for you cannot by that means combat the frothing of the liquor, or prevent small particles of the zinc escaping from the central tube into the acid liquor without, and so liberating and losing the arseniuretted gas. Again: to be certain of decomposing this gas, the exit pipe must be heated from the commencement, and if care has not been taken to expel the air, it will assuredly explode. Moreover, there is tact required in the application of the heat, or we either get the tube softened and bending, and perhaps blowing out, or the arsenium, by the great heat, is carried over, or else the gas is not decomposed at all; and if it so happen that the tube is a little too small, or gets closed up by a film of arsenium, the gas then bubbles through the acid liquor and is lost, and we cannot make a quantitative analysis: all these are difficulties in the way even of the experienced, but they are most formidable to the novice.

In place of this process, which I have long given up, I adopt the following, which is a modification of Lassaigne and Reinsch, and I never find the least difficulty in detecting even very small quantities of arsenic. Supposing I have the contents of a stomach to test. They are first boiled in distilled water, slightly acidulated with acetic acid, then filtered, and again and again boiled and filtered; mix these liquors and divide into two equal parts. Evaporate one part nearly to dryness, and then heat with about twice its bulk of sulphuric acid, until it is quite charred, and begins to evolve the acid. This mixture is then to be diluted and introduced, by little and little, into a large Wolfe's bottle, from which hydrogen is being slowly evolved from pure zinc and dilute sulphuric acid. The gas is to be conveyed by means of a bent tube, drawn fine at its remote point, to the bottom of a tall glass, containing a solution of nitrate of silver; it will occasion a black precipitate in it, and by taking the precaution of not allowing the gas to come over too quickly, and by means of the fine tube to make it pass through in very small bubbles, we shall be certain to decompose the whole of it. To this black turbid solution of nitrate of silver is then to be added muriatic acid, until all the silver is precipitated, and a little of the acid remains in excess; boil for a few minutes, and filter and evaporate the filtered liquor to dryness; the residue, if there be any, is to be dissolved in a little distilled water, and carefully precipitated by ammoniacal nitrate of silver; if any arsenic had been present, it would by this process have been converted into arsenic acid, and this with the ammoniacal nitrate would produce the red precipitate of arseniate of silver, every 464 grs. of which is equivalent to 100 grs. of arsenious acid, or 76 of arsenicum. This arseniate of silver may be again reduced by means of black flux, or charcoal, and preserved for evidence. The other half of the suspected liquor is to be slightly acidulated with muriatic acid, and boiled for an hour or two with a known weight of clean copper in strips; if arsenic is present, it will attach itself to the metal, giving it a black coating. The copper being removed, dried, and weighed, indicates how much heavier it may become, and to be certain of this being arsenic, they should be placed in a small green glass tube, closed at one end, and by means of a spirit lamp, with a large flame or a blow-pipe, kept at a red heat for a little time; if arsenic be there, it will sublime either as a white or black ring. After this, the copper is to be again weighed, and its loss estimated.

Supposing, however, that the substance to be tested were a piece of muscle or liver. It is to be charred by means of sulphuric acid, as in the first case, and the liquor diluted and divided into two portions; the one to be treated by Lassaigne's process with the nitrate of silver; the other to be nearly neutralized by carbonate of soda, and boiled, as in the last case, with the clean copper.

Now the advantages of these processes are, that they are readily applicable and will detect small quantities. The frothing is obviated, and you are not likely to be annoyed by the fallacies which antimony may occasion. Not but that antimony will decompose the nitrate of silver, and be deposited on the copper like arsenic; but then the subsequent stage of the processes determines this.

In conclusion, I may mention as a caution, that there is again in the market a large quantity of sulphuric acid contaminated with arsenic; I have found, in a recent sample, as much as one grain in the fluid ounce.—*London Lancet.*

